# Enduring Luminescence: Water and Heat Stable Perovskite Films via Hierarchical Hydrophobic Encapsulation

**DOI:** 10.1021/acsomega.5c08653

**Published:** 2025-11-20

**Authors:** Irem Tugce Aydemir, Kübra Ozkan Hukum

**Affiliations:** † Faculty of Science, Department of Chemistry, 37511 Gazi University, Ankara 06560, Turkey; ‡ Graduate School of Natural and Applied Sciences, 37511 Gazi University, Ankara 06560, Turkey

## Abstract

Lead halide perovskite nanocrystals have emerged as highly promising materials for optical devices due to their high photoluminescence quantum yield, excellent color purity, and low stimulated emission thresholds. However, one of the significant challenges limiting their practical application is the instability of nanocrystal films under various environmental conditions and elevated temperatures. In this study, we present a stabilization strategy involving the deposition of CsPbBr_3_ nanocrystals onto a template composed of a silica layer grown on a low-cost, soot-derived carbon skeleton, followed by hydrophobic encapsulation using polydimethylsiloxane (PDMS). The resulting thin films exhibited hydrophobic characteristics with a water contact angle exceeding 146.8°, retained high photoluminescence stability for up to 40 days in ambient air and 36 days under water, and demonstrated thermal resistance up to 100 °C. The incorporation of PDMS created a reliable moisture barrier, greatly boosting the perovskite layer’s resistance to environmental degradation. The results demonstrate that our approach offers a viable strategy for developing long-lasting and environmentally robust coatings for perovskite-based devices.

## Graphical abstract

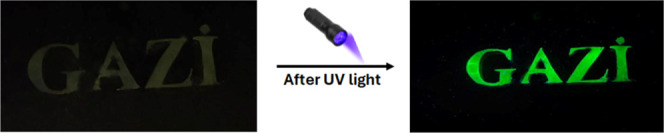

## 1 Introduction

Lead halide perovskite nanocrystals (LHP NCs) have attracted significant attention in recent years due to their exceptional optoelectronic properties, including narrow spectral bandwidth, high photoluminescence quantum yield, easily tunable emission wavelengths, high color purity, and low-cost processability.^1–4^ In particular, all-inorganic CsPbBr_3_ perovskite stands out for its strong photoluminescence in the green spectral region and relatively high thermal stability, making it highly promising for applications such as light-emission diodes (LEDs)^5^ and imaging systems. Nevertheless, despite these advantages, the poor environmental stability of LHP NCs remains a significant barrier to their widespread practical use. Exposure to environmental factors such as moisture, oxygen, and elevated temperatures can lead to degradation of the crystal structure, resulting in decreased photoluminescence efficiency and compromised structural integrity. These instabilities are primarily attributed to intrinsic factors, including the ionic nature of perovskites,^6^ low lattice energy,^7^ and weak adsorption–desorption equilibrium of surface ligands.^8^

Various encapsulation strategies have been developed to address these challenges and protect LHP NCs from environmental degradation. In particular, using inorganic matrices such as Al_2_O_3,_ ZnS, TiO_2_, and SiO_2_ to form physical barriers has been reported as an effective approach to enhance the resistance of LHP NCs against exposure to air and water.^9–13^ Similarly, polymer matrices and metal–organic frameworks (MOFs) have also been employed for stabilization purposes, aiming to achieve high photoluminescence efficiency and long-term durability. However, polymers such as poly­(methyl methacrylate) (PMMA) exhibit limited hydrophobicity, typically with a static water contact angle below 90°, which restricts their ability to provide complete protection against moisture ingress.^8^

At this point, integrating superhydrophobic surfaces with perovskite structures has emerged as a promising strategy for enhancing environmental stability. In the literature, Li and co-workers have demonstrated the fabrication of superhydrophobic surfaces on sponge substrates by employing candle soot (CS) and hydrophobic SiO_2_ nanoparticles.^14^ Similarly, Cao and colleagues applied CS-based coatings onto stainless steel surfaces, achieving high water repellency and super oleophilic characteristics. These structures demonstrated effective performance in oil–water separation applications.^15^ However, in many studies, the mechanical durability, long-term stability, and optical transparency of superhydrophobic structures remain inadequate. Moreover, most of these studies do not offer integrated solutions with perovskite structures, directly impacting optoelectronic performance. For instance, some reports involving coatings such as PMSQ on CsPbBr_3_ nanocrystals have demonstrated stability for up to 15 days under air and water exposure, yet their thermal resistance is typically limited to around 75 °C.^16^ Hu et al. Combined perovskite quantum dots with anhydrous silica spheres to enhance water and thermal stability. Their approach resulted in a photoluminescence (PL) retention of approximately 73.8% after 12 h under water exposure and about 36.8% after 15 h at 60 °C.^17^

In this context, the approach developed in this study distinguishes itself clearly from previously reported methods. A carbon-based template was fabricated using low-cost and nontoxic candle soot (CS), which provides a hierarchical micro/nanostructured surface essential for achieving super hydrophobicity. The subsequent growth of a silica skeleton rendered the structure mechanically robust, upon which photoluminescent CsPbBr_3_ nanocrystals were deposited to impart optical functionality. Finally, a PDMS coating was applied, forming a strong water-repellent barrier that significantly enhanced luminescence stability. Compared to similar reports in the literature, this multilayered architecture exhibited remarkable photoluminescence retention, maintaining stability for 40 days in air and 36 days under water exposure. Furthermore, it retained thermal durability up to 100 °C, outperforming previously reported systems in terms of longevity and functional reliability. These results demonstrate that the presented method offers a substantial advancement over existing strategies, combining functionality and sustainability to enhance the environmental resilience of perovskite-based optoelectronic devices.

## 2 Experimental Section

### 2.1 Materials

Lead­(II) bromide (PbBr_2_, ≥98%), oleylamine (OAm,70%), cesium carbonate (Cs_2_CO_3_, 99%), 1-octadecene (ODE, 90%), oleic acid (OA, 90%), tetraethyl orthosilicate (TEOS, 98%), ammonia (NH_3_), and ethanol (EtOH) were purchased from Sigma-Aldrich. PDMS ((C_2_H_6_OSi)­n, Sylgard 184), Sylgard 184 silicone elastomer curing agent, and paraffin wax were obtained commercially.

### 2.2 Synthesis of CsPbBr_3_ NCs

Lead halide perovskite nanocrystals (LHP NCs) were synthesized via the hot-injection method, following established protocols reported in the literature. In the first step, prepared a cesium oleate precursor solution. Cesium carbonate (Cs_2_CO_3_, 1 mmol, 350 mg) was placed in a 50 mL three-neck round-bottom flask, followed by the addition of 10 mL of 1-octadecene (ODE) and 1.5 mL of oleic acid (OA) under vacuum and nitrogen atmosphere. The reaction mixture was stirred at 400 rpm and heated to 120 °C under alternating vacuum and nitrogen until achieved complete dissolution of Cs_2_CO_3_.

In the second step, PbBr_2_ (54 mg) was placed into a 50 mL three-neck round-bottom flask, followed by the addition of 1 mL oleylamine (OAm), 1 mL oleic acid (OA), and 5 mL 1-octadecene (ODE). The mixture was stirred at 400 rpm and heated to 150 °C for 1 h to dissolve the PbBr_2_ completely. Subsequently, 1 mL of the previously prepared cesium oleate solution was swiftly injected into the hot solution. The reaction mixture was immediately quenched in an ice bath 5 s after injection. The resulting LHP NCs were precipitated using acetone and collected by centrifugation, followed by redispersion in hexane.^18^ All experiments were performed under a vacuum and nitrogen atmosphere to prevent moisture and oxygen exposure (Scheme 1).

**Scheme 1:**
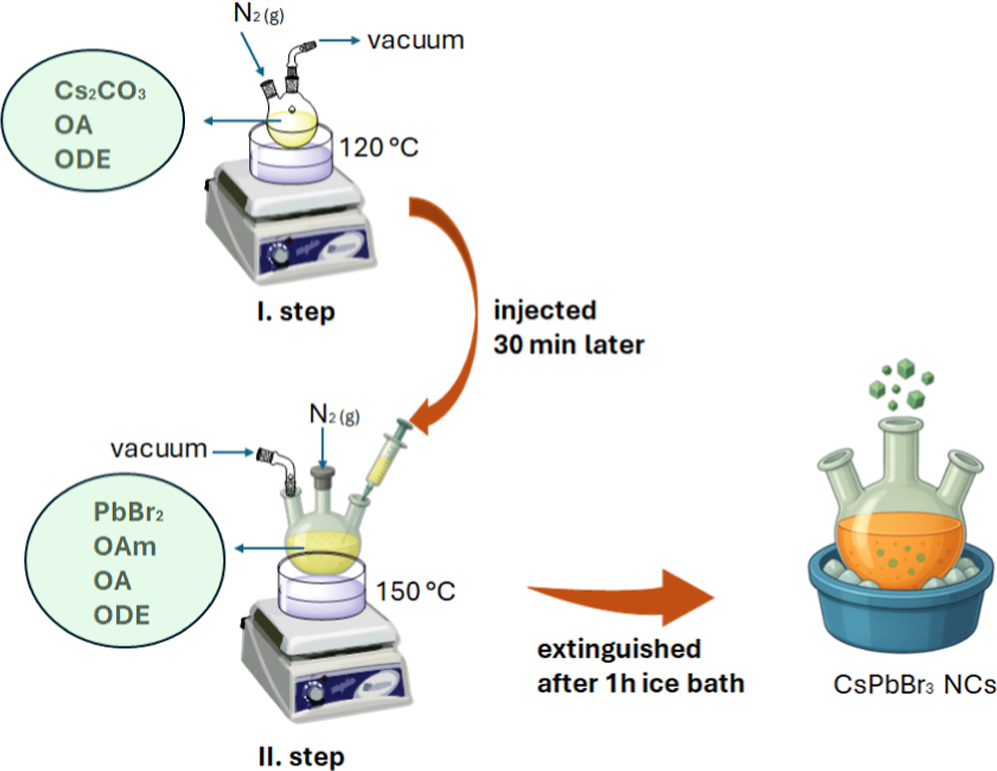
Schematics of the Hot-Injection Synthesis of CsPbBr_3_ NCs

### 2.3 Preparation of PDMS-Coated CsPbBr_3_ on Candle Soot

Glass slides were first cleaned in an ultrasonic bath with ethanol for ∼10 min and then dried. Subsequently, the glass surfaces were held 3 cm above the upper half of a candle flame for four different durations (30, 60, 90, and 120 s) to investigate the effects of CS particle deposition time on the morphological properties of the coated surfaces.

The black-coated glass slides were placed inside a vacuum desiccator, along with 1 mL each of tetraethyl orthosilicate (TEOS) and ammonia (NH_3_), which were separately introduced into the chamber. The system was maintained under vacuum at room temperature for 24 h. After, the surfaces were calcined at 600 °C for 2 h to achieve optical transparency. Upon completion of the calcination process, the resulting transparent surfaces were uniformly coated with CsPbBr_3_ NCs via spray deposition at a pressure of approximately 1–2 bar and a nozzle-to-surface distance of 10–15 cm.

In the final step, a homogeneous PDMS solution was prepared by mixing 10 g of PDMS base with 0.1 g of curing agent. A 2 mL portion of this solution was spin-coated onto the CsPbBr_3_-coated and blank glass surfaces at a constant spin speed of 1000 rpm for 30, 60, 90, and 120 s to investigate the film thickness variation with spin time. Further examined the effect of spin speed on film thickness by performing spin coating at speeds ranging from 1000 to 6000 rpm for a fixed duration of 120 s. Each prepared sample was placed in a glass Petri dish, covered with aluminum foil, and cured at 60 °C for 2.5 h. The resulting coating thicknesses were determined by cross-sectional SEM imaging (see Supporting Information, Figures S3 and S4). Additionally, fluorometric measurements were conducted on samples prepared at different spin speeds to evaluate the effect of coating thickness on photoluminescence intensity.

### 2.4 Characterization and Measurements

PL measurements of the CsPbBr_3_ NCs and film were performed using an Edinburgh Instruments (FLSP920) fluorescence spectrometer. The system was equipped with an Xe900 continuous xenon lamp (200–900 nm) for steady-state PL excitation. The morphology and size of the particles were examined using a FEI Tecnai G2 Spirit Biotwin high-contrast transmission electron microscope (CTEM) operated at 120 kV. The XRD patterns of the samples were determined by powder X-ray diffraction analysis using a Bruker D-8 ADVANCE diffractometer. Measurements were conducted in the 2θ range of 10°–50° under ambient conditions. Wettability was assessed via contact angle measurements of 2 μL deionized water droplets using a drop shape analysis system (DSA100, Krüss, Germany). Surface morphology and film thickness were characterized using a scanning electron microscope (SEM, HITACHI SU1000, Flex SEM 1000II) operated at 20 kV. Transmittance and absorption measurements were performed using the Jasco V770 UV–visible spectrophotometer. Additionally, to evaluate the film’s durability, a tape-peel test was conducted by repeatedly applying and removing 3 M Scotch adhesive tape from the surface at a 90° angle. The test was carried out for 10 cycles and measured the water contact angle after each peeling step.

## 3 Results and Discussion

CsPbBr_3_ NCs exhibiting strong green fluorescence were synthesized, where reaction temperature and time were critical parameters for controlling the emission wavelength and morphology during the reaction process. Structural characterization of the purified CsPbBr_3_ NCs via XRD confirmed that the synthesized CsPbBr_3_ adopted a cubic phase (PDF#54–0752). According to these results, the characteristic peaks observed at 2θ = 15.1°, 21.4°, 30.5°, 37.7° and 43.5° correspond to the (100), (110), (200), (211), and (220) crystal planes, respectively, based on the standard cubic CsPbBr_3_ NCs phase card (PDF#54–0752) (Figure 1a).^19,20^

**Figure 1:**
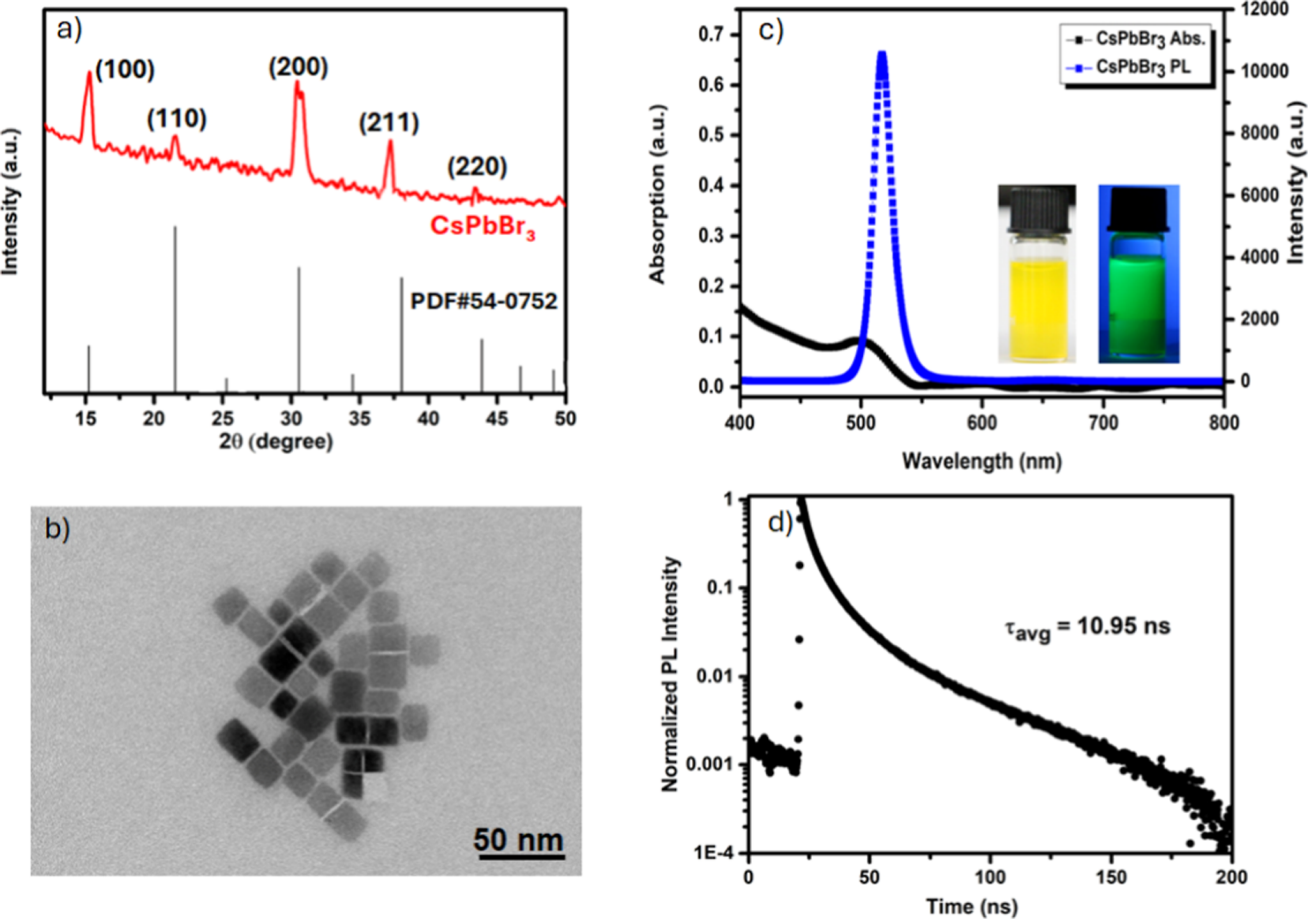
Synthesized CsPbBr_3_ NCs: (a) XRD patterns; (b) PL (blue) and UV–vis absorption spectra (black) with photographs of CsPbBr_3_ NCs solutions under daylight (left) and ultraviolet illumination (right); (c) TEM image (scale bar: 50 nm); and (d) time-resolved PL decay curve.

The crystal phase of CsPbBr_3_ NCs generally depends on the growth temperature.^21^ Growth temperatures exceeding 130 °C lead to the formation of a cubic phase, while temperatures below this threshold favor the formation of orthorhombic or monoclinic crystal structures. Figure 1b shows high-resolution TEM images of the CsPbBr_3_ NCs, exhibiting well-defined cubic morphology with an average size of 12.3 ± 1.7 nm (see Supporting Information, Figure S1). The optical properties of the synthesized CsPbBr_3_ NCs were investigated using UV–vis and fluorescence spectroscopy. As presented in Figure 1c, the CsPbBr_3_ NCs exhibit absorption (black) and emission (blue) spectra. A clear absorption band edge was observed at approximately 500 nm in the UV–vis spectrum. Under 380 nm excitation in hexane, the emission spectrum displays a narrow excitonic band with a maximum at 520 nm.

Furthermore, to analyze the PL Dynamics of this sample, time-resolved PL decay curves were recorded using a 380 nm pulsed laser as the excitation source. The resulting decay profiles, shown in Figure 1d and Table 1, can be accurately fitted with a triple-exponential function. The average lifetime of the synthesized CsPbBr_3_ NCs was determined to be 10.95 ns. The fitted decay curves revealed three characteristic time constants, τ_1_, τ_2_, and τ_3_, indicating the presence of multiple emissive centers with different recombination rates in the sample.^22^ As previously reported, τ_1_ is attributed to excitation recombination involving surface states and defects, τ_2_ is assigned to radiative recombination, and τ_3_ is associated with nonradiative recombination. The amplitude parameters *A*_1_, *A*_2_, and *A*_3_ are considered weighting factors.

**Table 1:** Fitted Lifetimes of the CsPbBr_3_ NCs

sample	*A*_1_	τ_1_ (ns)	*A*_2_	τ_2_ (ns)	*A*_3_	τ_3_ (ns)	Τ_avg_
CsPbBr_3_	0.30	1.88	1.65	5.21	4.45	13.70	10.95

The final morphological structure and wettability characteristics of the surface are highly dependent on the specific process parameters and material deposition conditions. In this study, observed that the properties of the resulting coating varied significantly with both the processing procedure and the chemical vapor deposition (CVD) duration of TEOS. These two parameters critically influence nanoscale surface architecture, thereby playing a key role in determining whether the sample exhibits hydrophobic or hydrophilic behavior. In particular, candle soot deposition time and height variations enabled fine-tuning of the template morphology.^23^ Initially, candle soot (CS) surfaces were prepared using a practical and straightforward method. Glass slides were held above a candle flame at approximately 3 cm for durations ranging from 30 to 120 s, during which soot particles were deposited onto the glass surface (see Supporting Information, Figure S2a–c). The simplicity of this method allows for controlled morphology formation. The mass of the accumulated deposit was determined by weighing the glass slides before and after the accumulation process; a linear increase in mass was observed depending on the accumulation time (see Supporting Information, Figure S2d). When held above the flame, the glass slides acted as a barrier between the flame and ambient oxygen, creating incomplete combustion conditions that facilitated the formation of carbon nanoparticles.^24^ As observed in the SEM images (Figure 2a), the carbon-based candle soot particles formed an irregular morphology consisting of interconnected dendritic fractal structures with high surface roughness. SEM images of soot layers deposited at different durations revealed no significant changes in surface topography. In the second step, these soot-coated surfaces were treated with TEOS, forming silica particles on the surface. At this stage, a more uniform coating was observed compared to the bare candle soot, indicating the formation of silica shells (Figure 2b). Due to the inherently hydrophilic nature of silica, the subsequently deposited CsPbBr_3_ nanocrystals adhered more densely and uniformly to the surface, forming a rougher and more continuous luminescent layer (Figure 2c). In the final step, the surfaces were coated with PDMS at a spin speed of 6000 rpm, resulting in a layer with a thickness of approximately 6.1 μm. As reported in previous studies, this process led to the emergence of a buckled microstructure (Figure 2d). The observed buckling is believed to originate from stress-induced pressure within the deposited multilayer. The PDMS film appears to be unable to withstand the interfacial stress forces at the boundary between the PDMS and the underlying material, leading to surface expansion and the development of a wrinkled or buckled topography.^25^ The effect of surface modifications on wettability was evaluated through static water contact angle measurements. The contact angles measured after each modification step clearly reflected the changes in surface properties (Figure 2e). Initially, the pristine CS-coated surface exhibited a high-water contact angle of 150.6°, causing water droplets to roll off easily. In contrast, the surface covered with silica particles displayed hydrophilic behavior, with the contact angle decreasing to 20.5°. After deposition of CsPbBr_3_ nanocrystals, the contact angle increased to 88.5°, and upon final PDMS coating, it reached 146.8°. These results indicate that the presence of alkyl groups in PDMS, combined with the microscale wrinkled morphology, significantly enhanced the hydrophobicity of the surface. Previous studies have also shown that hydrophobic surface characteristics in perovskite-based structures directly influence device performance, highlighting the importance of improved water resistance and stability under humid conditions for prolonging device lifetime and maintaining efficiency.^26^

**Figure 2:**
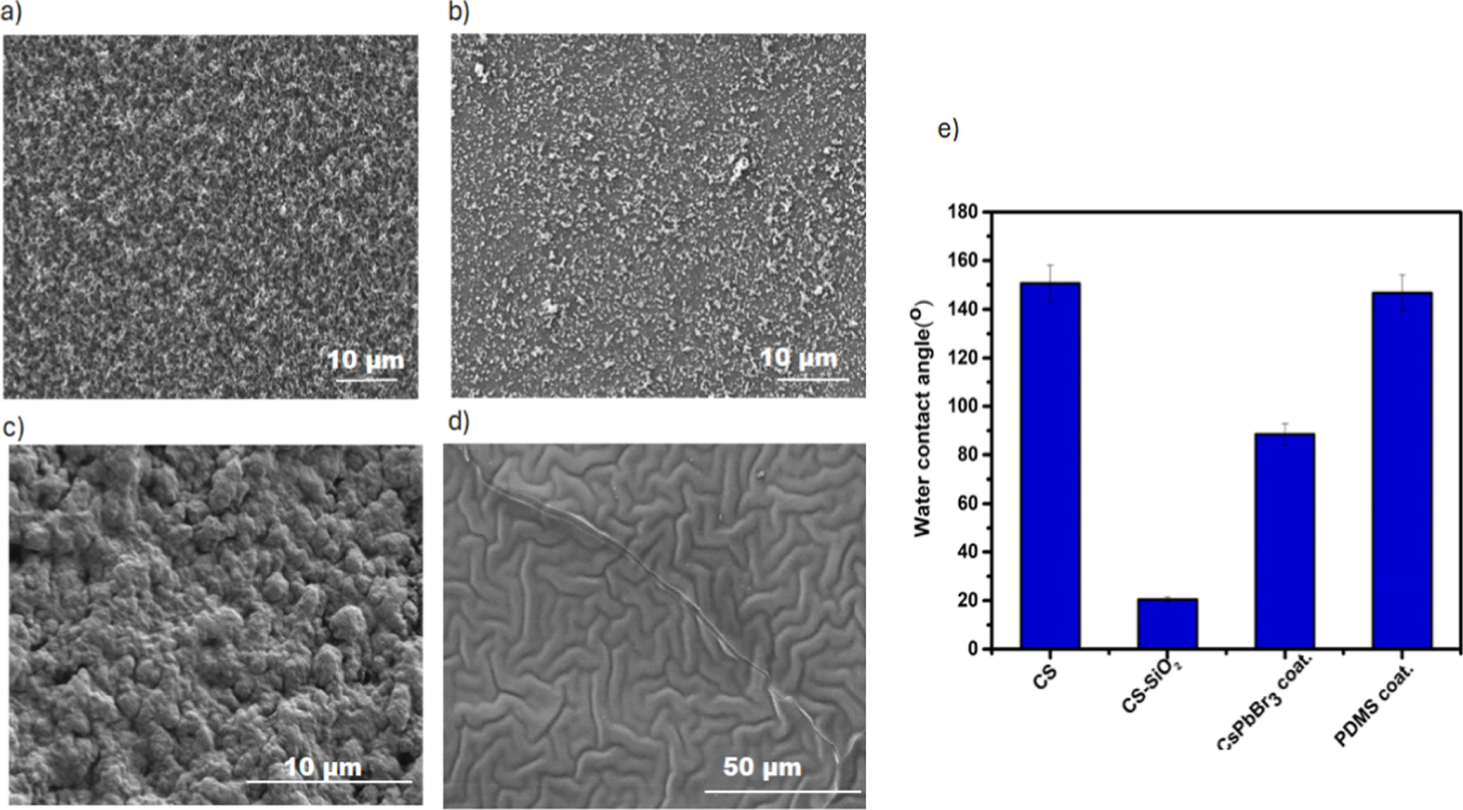
SEM images of (a) candle soot, (b) silica particle-coated, (c) CsPbBr_3_ NCs, and (d) PDMS-coated glass surfaces; (e) static water contact angle measurements at each modification step.

Considering the physical properties of PDMS during the design process, a spin-coated technique was employed to deposit it onto CsPbBr_3_-coated substrates. Although this coating method is relatively simple, achieving a uniform film with optimized sensitivity requires the use of appropriate spin durations and speeds. These two parameters are critical for attaining the desired film thickness on the substrate. As shown in Figure 3a, under a constant spin speed of 1000 rpm, the film becomes thinner and more uniform as the spin duration increases, indicating that film thickness is influenced by spin time (see Supporting Information, Figure S3). However, with respect to spin speed, film thickness is inversely proportional. Theoretically, as supported by the results, increasing the spin speed leads to a gradual decrease in film thickness.^27^ The thickest PDMS film (94.4 μm, see Supporting Information, Figure S4i) was obtained at the lowest spin speed of 1000 rpm. As the spin speed increased, thinner films were achieved, with the thinnest layer (6.1 μm, see Supporting Information, Figure S4vi) recorded at the highest spin speed used in this study, 6000 rpm. In the early stage of spin coating, the PDMS solution behaves as a viscous liquid, allowing it to spread uniformly across the substrate surface under the influence of centrifugal force. This viscous flow not only facilitates the formation of a uniform film but also enables the excess solution to be removed from the substrate. At higher spin speeds, the centrifugal force increases, expulsing a greater amount of PDMS solution from the surface. Consequently, this leads to a reduction in the final thickness of the PDMS film. Figure 3b illustrates the variation in film thickness as a function of spin speed. The error bars represent the maximum and minimum thickness values measured at different points on each sample, while the circular markers indicate the average thickness. Higher spin speeds, which are associated with stronger centrifugal forces during the spin-coating process, result not only in thinner films but also in improved uniformity. In addition, the optical properties of PDMS and PDMS@CsPbBr_3_ materials were investigated using UV–vis absorption, transmittance, and photoluminescence (PL) spectroscopy (see Supporting Information, Figures S5 and S6). Bare PDMS films exhibited high transmittance exceeding 90% in the visible region (Figure 3c), while a slight decrease was observed at short wavelengths as film thickness increased (see Supporting Information, Figure S5a). This is due to increased light scattering due to internal reflection in thicker layers. The almost negligible absorption in the visible region and the absence of photoluminescence emission confirm that PDMS is an optically transparent and passive material (see Supporting Information, Figure S5b,c). On PDMS@CsPbBr_3_ surfaces, a general decrease in film transmittance was observed upon incorporating CsPbBr_3_ perovskite particles into the PDMS matrix (Figure 3c). This decrease was particularly pronounced in thicker films and is thought to be due to increased light scattering and perovskite-induced absorption with increasing film thickness. In the absorption spectrum, the absorption peak around 520 nm, corresponding to the characteristics band edge of CsPbBr_3_ perovskites, was observed to decrease as the spin speed increased. This demonstrates that film thickness has a direct effect on optical density (see Supporting Information, Figure S6). In Figure 3d, the room-temperature photoluminescence (PL) spectra of the bare CsPbBr_3_ film and PDMS-coated samples at different spin speeds (1000–6000 rpm) are compared. All samples were measured under identical excitation conditions using a fixed excitation wavelength of 380 nm. The bare film (black curve) exhibited the highest PL intensity, with a prominent emission peak centered around 519 nm. As significant decrease in PL intensity and a slight blue shift were observed as the thickness of the PDMS layer increased. In particular, the sample coated at 1000 rpm (with a film thickness of 28.8 μm) showed nearly a 50% decrease in PL intensity. These effects may be attributed to the optical properties of PDMS (such as refractive index mismatch, light scattering, and absorption) as well as possible internal stress effects within the multilayer structure. On the other hand, a slight blue shift of approximately 3.5 nm was detected in the PL peak position because of PDMS coating. This minor shift may be attributed to changes in the dielectric environment or surface stress induced by the polymer layer. Similar magnitudes of blue shifts after polymer coating have been reported in other halide perovskite systems.^29^ This also shows that PDMS does not induce substantial deformation in the perovskite crystal structure but partially alters the surface environment.

**Figure 3:**
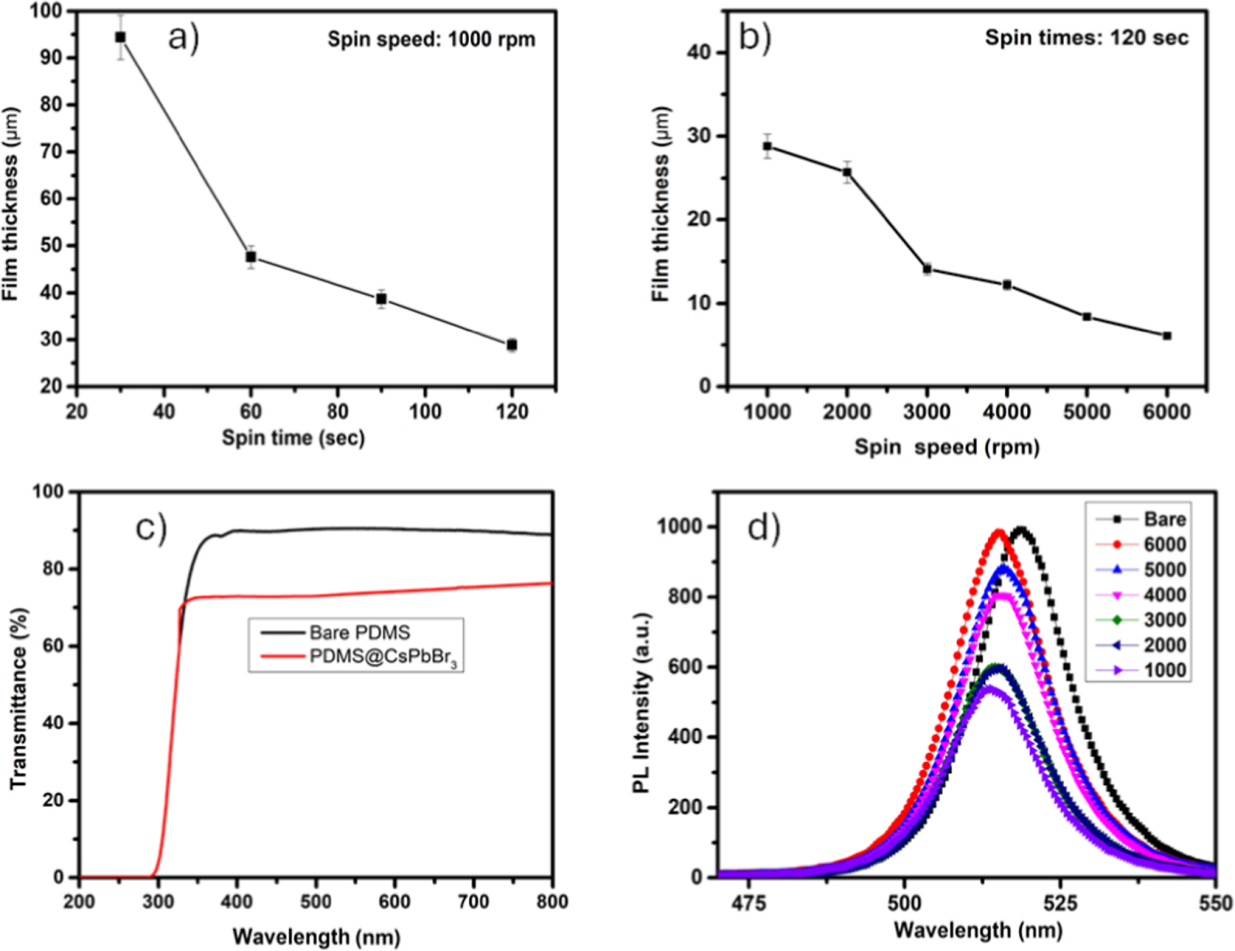
PDMS thicknesses at (a) a constant spin speed (1000 rpm) varying durations, (b) a fixed duration (120 s) varying spin speeds, (c) transmittance of a bare PDMS and a PDMS@CsPbBr_3_ coating, and (d) effect of PDMS thickness on the PL spectra of CsPbBr_3_ films.

Following the determination of optimum coating conditions, the thinnest and most homogeneous PDMS film obtained under these conditions (6000 rpm for 120 s) was subjected to aging tests to evaluate the environmental stability of the CsPbBr_3_ NC surfaces. As observed in the absorption spectrum, the hydrophobically passivated film exhibited significantly enhanced stability against moisture (Figure 4a). After 40 days of air aging, the photoluminescence (PL) intensity of the CsPbBr_3_ NC suspension retained ∼86% of its original value. This finding demonstrates that PL stability is positively correlated with surface hydrophobicity, which protects NCs from moisture-induced degradation. Furthermore, during 40 days of storage under ambient atmospheric conditions, no color change was observed in the hydrophobically passivated CsPbBr_3_ samples (Figure 4b). Remarkably, the passivated luminescent surfaces exhibited water-repellent behavior even under submerged conditions. In the absence of PDMS passivation, the samples degraded within 10 min of water exposure, exhibiting a sudden color change and a noticeable blue shift. In contrast, the PDMS-passivated surfaces maintained strong stability under water, exhibiting no color change and preserving their green emission for up to 36 days (Figure 4c,d). These findings confirm the strong water resistance of the fabricated films. The use of such a hydrophobic protective film is promising for significantly extending the operational lifetime of CsPbBr_3_ NC-based devices in aqueous environments.^28^ Nevertheless, CsPbBr_3_ NCs are not only highly sensitive to oxidation but also exhibit substantial instability at elevated temperatures. To investigate this, PL measurements were performed on PDMS-coated CsPbBr_3_ NCs under different temperature conditions, with each temperature maintained for ∼5 min. As shown in Figure 5, the PL efficiency gradually decreased to approximately 93%, 76%, 63%, 42%, and 30% as the temperature increased up to 100 °C. These results clearly demonstrate that the PL intensity of CsPbBr_3_ NCs declines drastically at higher temperatures. The primary reason for this pronounced decrease is the reduction in surface bonding induced by thermal stress, which accelerates the degradation process.

**Figure 4:**
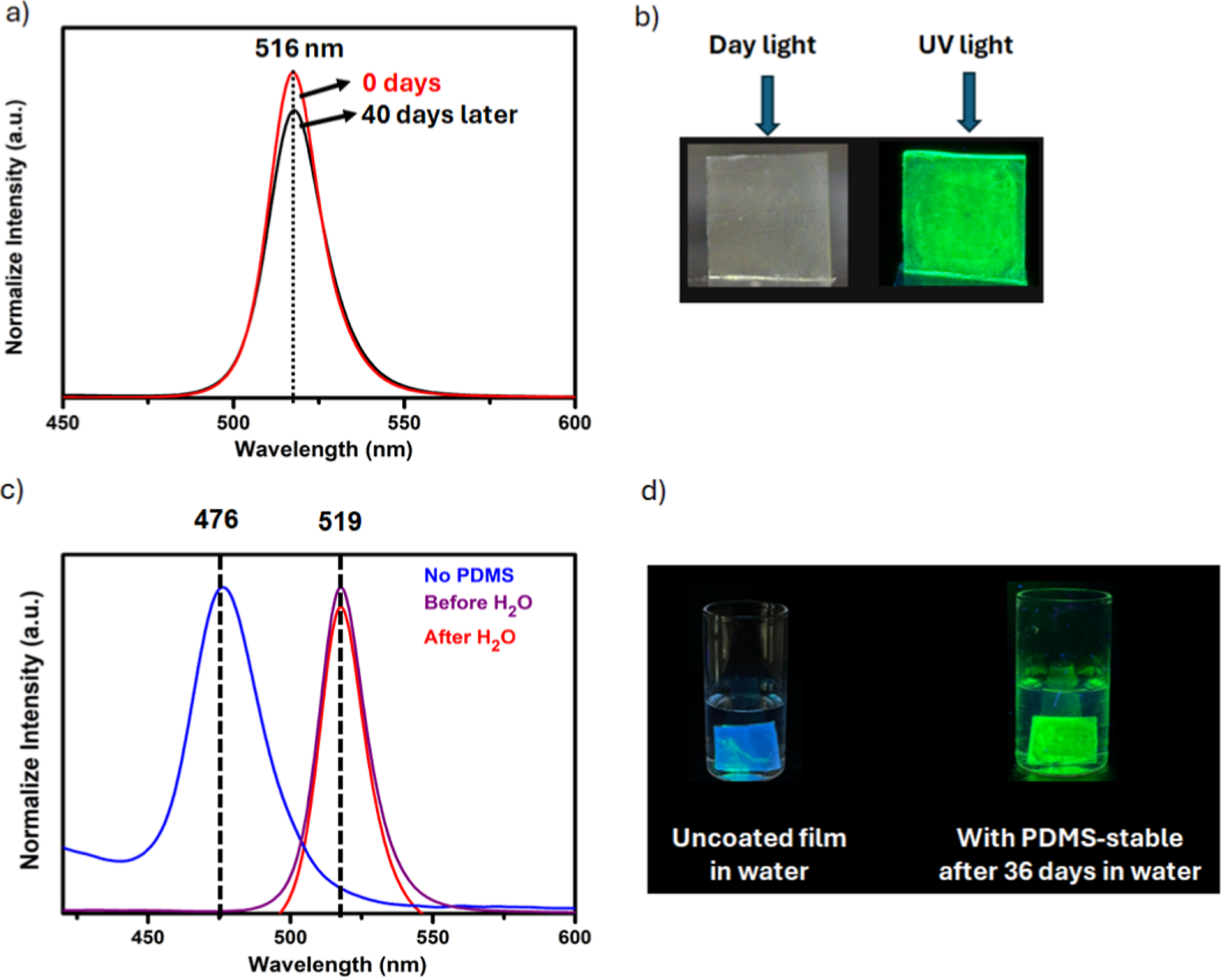
(a) PL spectra of PDMS-coated CsPbBr_3_ stored under ambient conditions (initial: red, after 40 days: black), (b) photographs of the surfaces under daylight and UV illumination, (c) PL spectra of PDMS coated (initial: burgundy, after 36 days: red) and uncoated (after 10 min: blue) surfaces in a aqueous environment, and (d) photographs of PDMS-coated and uncoated surfaces after specified duration.

**Figure 5:**
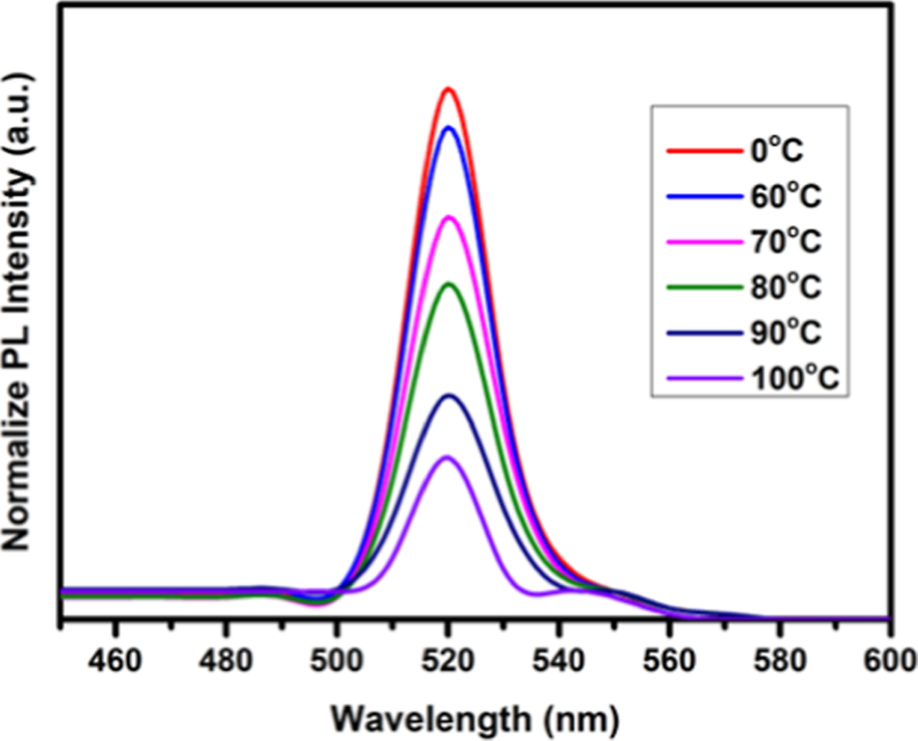
Temperature-dependent PL spectra of PDMS-coated CsPbBr_3_ surfaces.

To evaluate the adhesion of PDMS@CsPbBr_3_ films to glass substrates, a tape-peeling test was conducted. In this method, adhesive tape (3 M Scotch tape) was applied to the film surface and subsequently peeled off at a 90° angle. This procedure was repeated 10 times, and after each cycle, the water contact angle (WCA) on the films was measured (Figure 6). Remarkably, it was observed that the water contact angle of the film exhibited only minimal changes even after undergoing 10 cycles of the peeling test. Throughout the testing process, the film maintained its highly hydrophobic characteristics, demonstrating its strong adhesion to the substrate.

**Figure 6:**
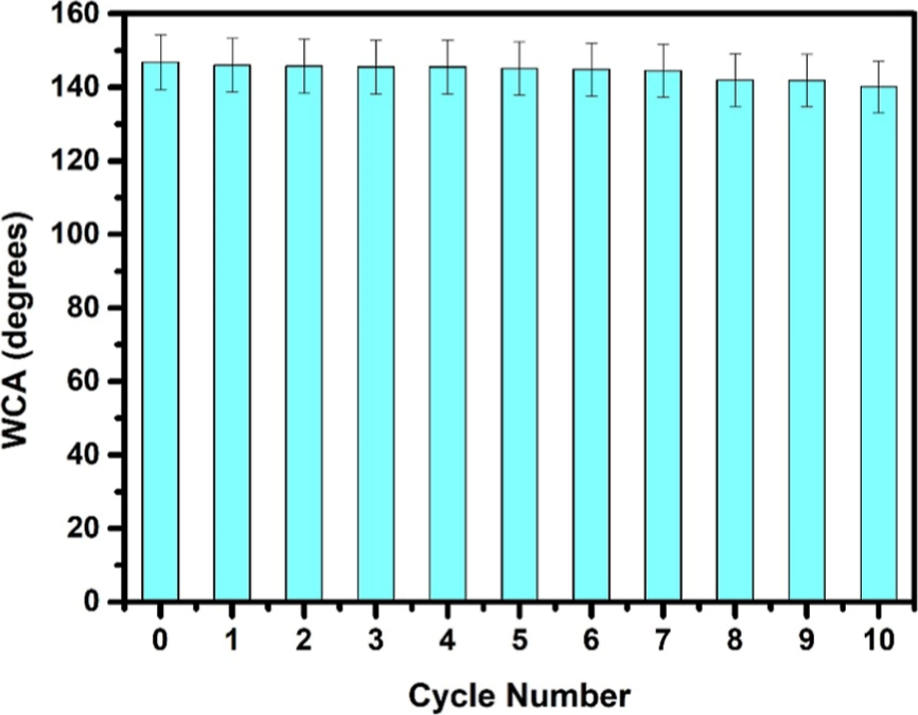
WCA on PDMS@CsPbBr_3_@CS film deposited glass substrate after peel-off test for 10 cycles.

## 4 Conclusions

The result presented in this study highlights the effectiveness of the developed multilayer encapsulation strategy in enhancing the environmental durability of CsPbBr_3_ nanocrystal (NC) films. Compared to existing encapsulation techniques (such as polymer composites (e.g., PMSQ/AG), inorganic shells (e.g., SiO_2_, Al_2_O_3_), and MOF-based systems), our approach, combining a hierarchical carbon-silica scaffold with a hydrophobic PDMS overlayer, offers significant improvements in water, air, and thermal stability. The superhydrophobic surface, achieved through candle soot as a carbon template and subsequent silica deposition, provided a highly textured micro/nanostructured morphology that facilitated water repellency (contact angle > 150°). While some literature reports have demonstrated water stability up to 30 days,^19^ the PDMS-passivated CsPbBr_3_ NC films in this work exhibited photoluminescence (PL) stability for 36 days under water immersion. Moreover, air exposure for 40 days resulted in only ∼14% PL loss, further exceeding stability benchmarks previously reported (typically ≤ 30 days). Thermal stability remains a critical limitation for perovskite-based materials. While earlier strategies achieved moderate thermal resistance (generally ≤75–80 °C),^16^ our films maintained significant PL intensity up to 100 °C, confirming the robustness of the PDMS layer under thermal stress. However, the absence of any spectral shift suggests preservation of the perovskite crystalline phase. Another key finding is the ability to tune PDMS film thickness via spin-coating parameters. Thicker films (>90 μm) were associated with PL attenuation, likely due to light scattering and refractive index mismatch, whereas thinner coatings (∼6 μm) provided an optimal balance between protection and optical transparency. Overall, the integration of microstructural engineering (via soot templating), chemical robustness (via silica), and surface hydrophobicity (via PDMS) enables a multifunctional and scalable platform for perovskite stabilization. This strategy demonstrates a promising route for extending the operational lifetime of perovskite-based optoelectronic devices under realistic environmental conditions.

## Supplementary Material



## Notes

Histogram of TEM particle sizes for the synthesized CsPbBr_3_ NCs. SEM images of candle soot deposited for 60, 90, and 120 s. Cross-sectional SEM images of films spin-coated at a fixed speed (1000 rpm) for varying durations. Cross-sectional SEM images of films spin-coated for a fixed time (120 s) at different spin speeds (1000–6000 rpm). Transmittance, absorption, and PL graphs of blank PDMS-coated glass surfaces. Transmittance, and absorption graphs of PDMS@CsPbBr_3_-coated surfaces (PDF)
