# Supplementary material for: Enduring Luminescence: Water and Heat Stable Perovskite Films via Hierarchical Hydrophobic Encapsulation
Source: ACS Omega. 2025 Nov 20;10(48):59282–9. doi: 10.1021/acsomega.5c08653 (PMC12771148; doi:10.1021/acsomega.5c08653)

SUPPORTING INFORMATION

Enduring Luminescence: Water and Heat Stable Perovskite Films via Hierarchical Hydrophobic Encapsulation

Irem Tugce AYDEMIR[1,2] and Kübra OZKAN HUKUM[1*]

[1] Gazi University, Faculty of Science, Department of Chemistry, Ankara, TURKIYE

[2] Graduate School of Natural and Applied Sciences, Gazi University, Ankara, TURKIYE

Email: kubraozkanhukum@gazi.edu.tr

Supporting Information

Figure S1.Particle size distribution histogram of $CsPbBr_3$ NCs

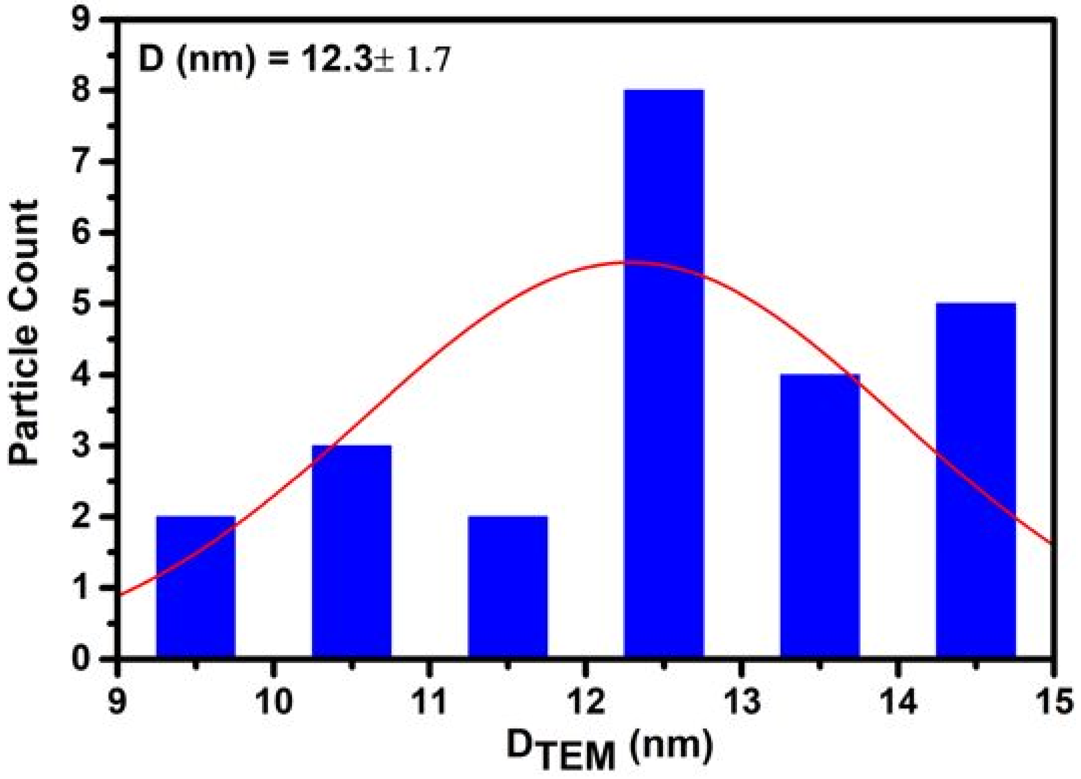

Figure S2. SEM images of candle soot deposited on glass substrates at 60 (a), 90 (b), and 120 s (c); amount of candle soot deposited at different deposition times (d)

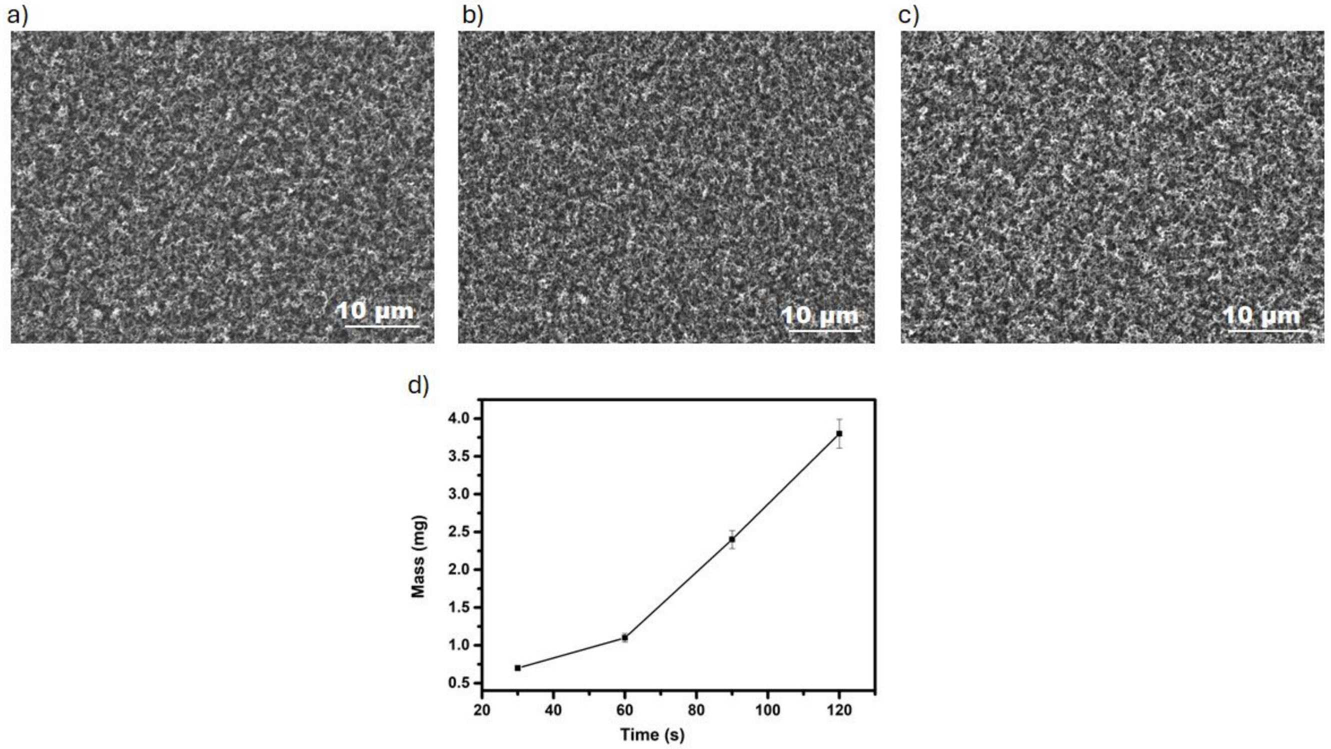

Figure S3. Cross-sectional SEM image of the composite film coated on the substrate at a constant speed of 1000 rpm for different times (i-30, ii-60, iii-90, and iv-120 sec.)

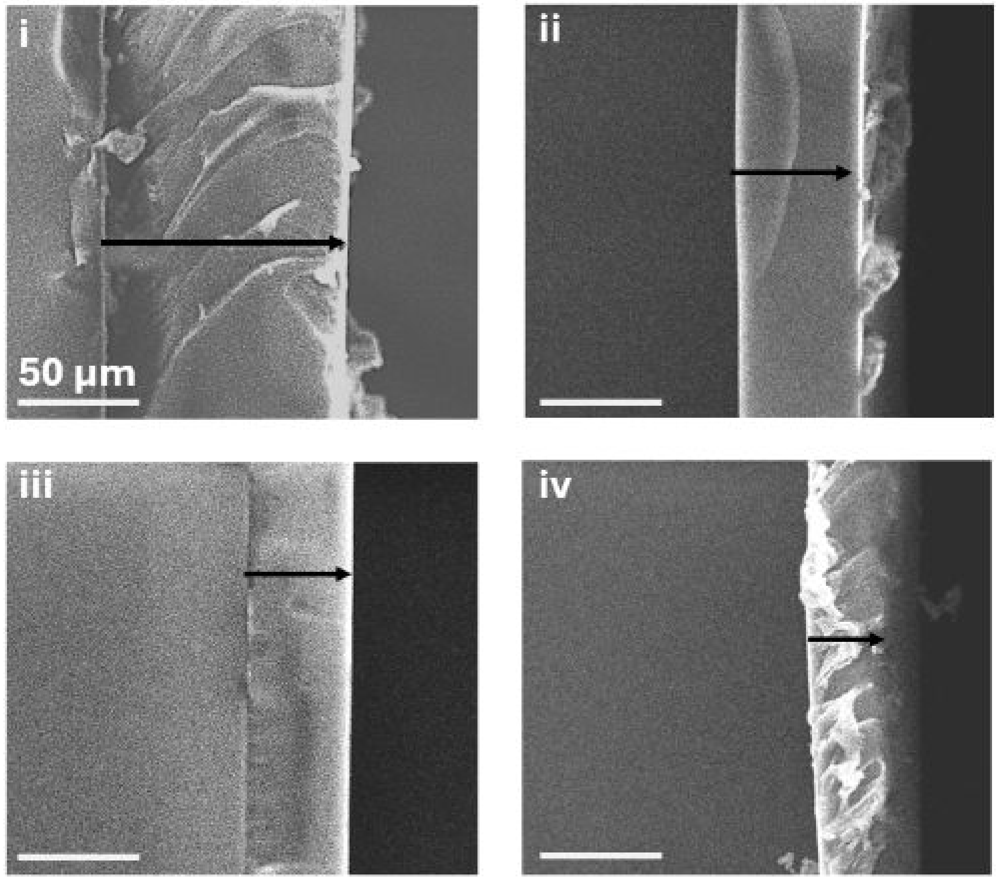

Figure S4. Cross-sectional SEM image of the composite film coated on the substrate at different speeds (i-1000, ii-2000, iii-3000, iv-4000, v-5000, and vi-6000 rpm) with a fixed time of 120 seconds

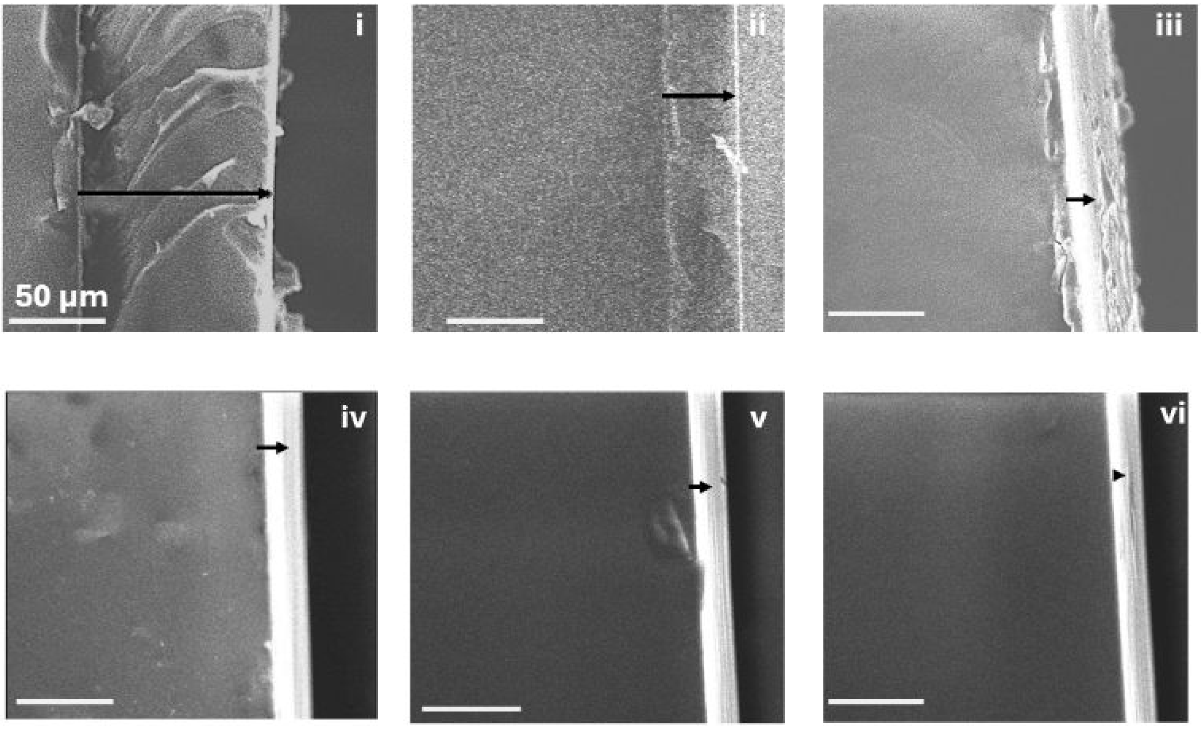

Figure S5. a- Transmittance, b- absorption, and c- PL graphs of blank PDMS-coated glass surfaces

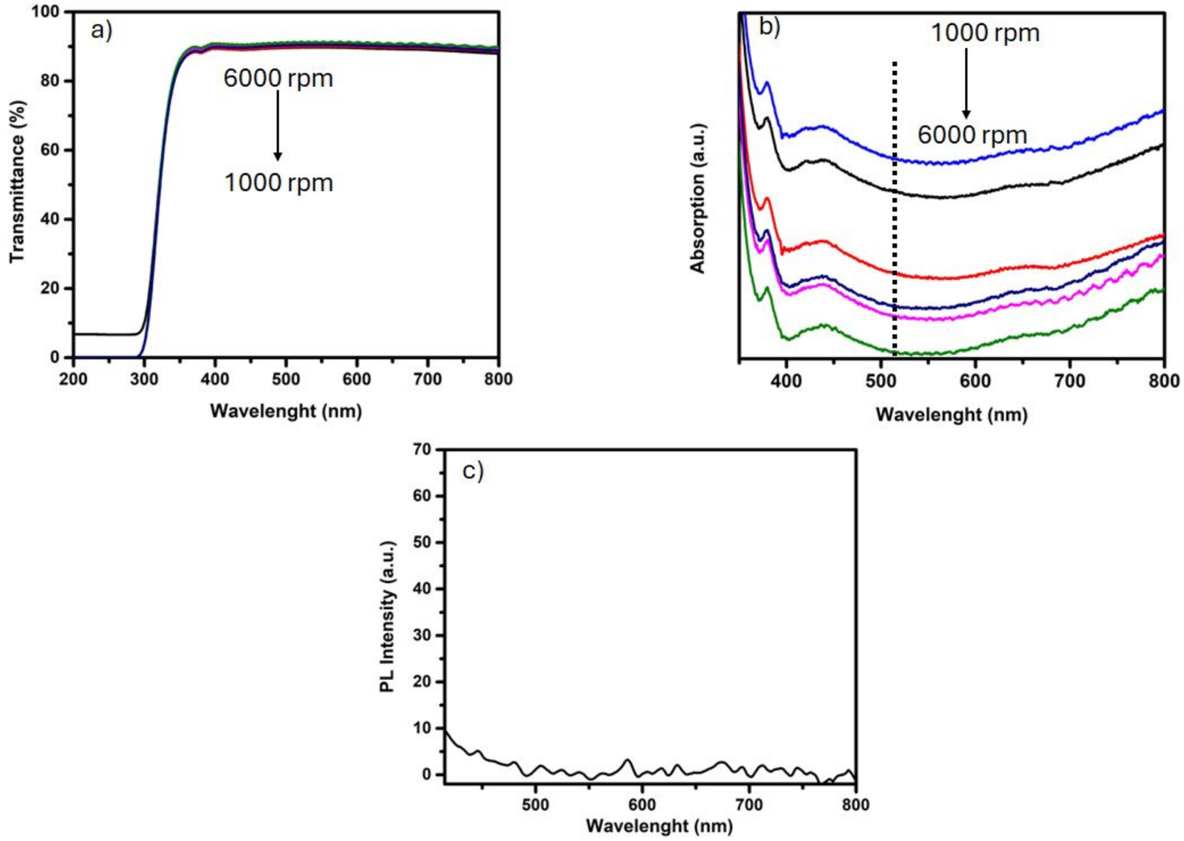

Figure S6. a- Transmittance, and b- absorption graphs of PDMS@$CsPbBr_3$ coated surfaces

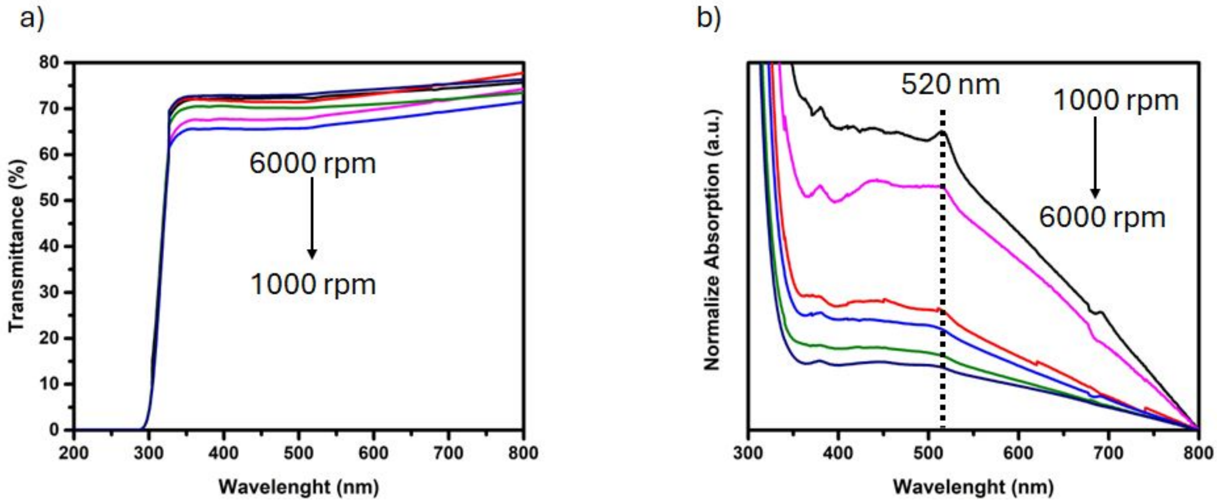

Supplement: Supplementary file 1 [file ao5c08653_si_001.pdf]
